# Challenges in diagnosis of clinical and subclinical *Plasmodium falciparum* infections in Ghana and feasibility of reactive interventions to shrink the subclinical reservoir

**DOI:** 10.1186/s12936-024-05096-6

**Published:** 2024-09-10

**Authors:** Madeline Reynders, Austine Tweneboah, Dawood Ackom Abbas, Stephen Opoku Afriyie, Stephen Nelly Nketsiah, Kingsley Badu, Cristian Koepfli

**Affiliations:** 1 Eck Institute for Global Health & Department of Biological Sciences, University of Notre Dame, Notre Dame, IN USA; 2 Department of Theoretical and Applied Biology, Kwame Nkrumah University of Science and Technology (KNUST), Kumasi, Ghana

**Keywords:** Malaria control, Diagnosis, Active case detection, Subclinical infection, Subpatent infection

## Abstract

**Background:**

Reactive case detection (RCD) aims to reduce malaria transmission stemming from asymptomatic carriers. Symptomatic individuals diagnosed with malaria at a health centre are followed to their households, where members of the index case and neighbouring households are tested and treated for malaria. An RCD programme was tested in the Ashanti region of Ghana in order to study diagnostic accuracy in the hospital and household settings, assess the prevalence of subclinical infections and possible clustering in index case households, and identify operational challenges for future RCD programmes. Currently, transmission in this region is high, but reactive interventions might become an option once transmission is reduced.

**Methods:**

264 febrile individuals were enrolled at the Mankranso Government Hospital and tested for malaria using rapid diagnostic tests (RDT). From the pool of RDT-positive febrile index cases, 14 successful RCD follow-ups were conducted, and 233 individuals were enrolled from the index case, neighbour, and control households. The sensitivity of diagnostic tools for clinical and subclinical cases was compared, including RDT, expert microscopy by World Health Organization-certified microscopists, field microscopy, and qPCR.

**Results:**

Poor diagnosis and low receptivity to RCD-style follow-ups were major limitations to a successful and effective RCD programme. Field microscopy detected only 49% of clinical infections compared to RDT. 54% of individuals did not agree to a follow-up, and 66% of attempted follow-ups failed. The system effectiveness of RCD, calculated as the product of correctly diagnosed index cases, successful follow-ups, and proportion of asymptomatic infections detected by RDT, was very low at 4.0%.

**Conclusions:**

Due to low system effectiveness and the endemic nature of the disease setting in which asymptomatic prevalence is high and infections are not clustered around index case households, RCD is currently not a feasible option for malaria control in this region. The operational challenges identified through this study may help inform future reactive intervention programme designs once transmission is reduced.

**Supplementary Information:**

The online version contains supplementary material available at 10.1186/s12936-024-05096-6.

## Background

In 2021, there were an estimated 247 million global malaria cases, with the World Health Organization (WHO) African Region accounting for approximately 95% of cases [1]. In endemic areas, subclinical infections might be the source of over 90% of transmission [2, 3]. Subclinical individuals have partial immunity to the malaria parasite, often resulting in lower parasite density. Interventions that shrink the subclinical reservoir are likely to be needed in order to make gains towards malaria control and eventual elimination.

Reactive case detection (RCD) programmes are an approach to reducing transmission by subclinical carriers based on the clustering of subclinical infections around detectable clinical infections [4]. After initial hospital diagnosis, health workers travel to the household of the symptomatic index case and test and treat members of the index case and neighbouring households to potentially eliminate further peri-domestic transmission from asymptomatic carriers [4]. A meta-analysis found that in low-transmission settings, there were approximately 4-fold greater odds of detecting infections in households where an individual with a symptomatic malaria case lives [5]. Where transmission is very low, testing and treatment of all members of index case households might eliminate 75% of infections in a community [5]. The effectiveness of RCD critically depends on proper diagnosis of clinical index cases at health centres and sensitive detection of subclinical secondary infections. As a major limitation of RCD programmes, rapid diagnostic tests (RDTs) typically used for diagnosis, often fail to detect low-density infections. Reactive focal drug administration (rFDA) overcomes this limitation. In contrast to RCD all members of index case and neighbour households are given anti-malarial drugs. Multiple studies have found that in low-transmission settings, rFDA programmes reduced malaria transmission and were concluded to be more effective, quicker, and easier to implement than RCD programmes [6, 7]. Alternatively, uniformly applied interventions, such as anti-malarial mass drug administration (MDA) and mass test and treat (MTAT) campaigns might be used to reduce the subclinical reservoir and interrupt transmission.

The WHO recommends focal drug administration (rFDA) and reactive case detection (RCD) only in regions nearing elimination, as else they are unlikely to any effect on malaria transmission [1, 8]. In addition to reducing interventions, reactive interventions can support surveillance, e.g. by providing data on changes in the prevalence of infections, risks factors, and spatial clustering of transmission. Ghana has shown consistent progress towards malaria control in recent years, with nationwide malaria parasite prevalence (by microscopy diagnosis) in children aged 6 to 59 months decreasing from 28% in 2011 to 14% in 2019 [9]. The Ghanian National Malaria Strategic Plan 2021–2025 has three main goals: reduce malaria mortality by 90%, reduce malaria case incidence by 50%, and achieve malaria pre-elimination in at least six districts [9]. Ghana has several malaria control programmes in effect, including indoor residual spraying (IRS) campaigns, insecticide-treated net (ITN) distribution, and intermittent preventative treatment in pregnancy (IPTp) programmes, but the country does not currently have any reactive case management programmes [9]. The CHPS (Community-based Health Planning and Services compounds) Initiative in Ghana provides RDTs, ACT (artemisinin-based combination therapy), and IPTp for pregnant women [9]. The malaria burden is not homogenous throughout the country; in 2019, 2% of children aged 6 to 59 months tested positive for malaria by microscopy in the Greater Accra region, yet 27% of children tested positive in the western region of Ghana [10]. The Greater Accra region may be a target region for the implementation of an elimination strategy due to the lower malaria incidence as compared to other regions of the country.

The current study followed up clinical patients in the Mankranso District in the Ashanti Region of Ghana, where reactive interventions have never been conducted. Asymptomatic cases in the Ashanti region likely contribute greatly to the overall malaria burden of Ghana; a 2020 study found that the molecular prevalence of asymptomatic *Plasmodium* infection was 73% [11]. This study evaluated the feasibility of reactive interventions in order to systematically collect data regarding diagnostic accuracy in the hospital and household setting using the latest generation of highly sensitive RDTs, determine whether clustering of infections is observed at the current high prevalence levels, and identify operational challenges that may impact the feasibility of future reactive interventions, including RCD or rFDA. Community-specific operational and implementation challenges faced in this study will help inform future reactive intervention programmes that will be critical when transmission is reduced and Ghana transitions to an elimination goal.

## Methods

### Ethical approval and community engagement

The study was approved by the Committee on Human Research, Publication and Ethics of the Kwame Nkrumah University of Science and Technology (KNUST), School of Medical Sciences and Komfo Anokye Teaching Hospital, and the University of Notre Dame IRB (approval no. 19-04-5321). All study participants or their parents or legal guardians provided informed written consent before sample collection.

The study was conducted at the Mankranso Government Hospital in Mankranso, a small, peri-urban community about 30 km outside of Kumasi, Ghana, in June and July 2022. This is a year-round high transmission disease setting. The KNUST study team had been working with Mankranso Hospital and surrounding communities since 2019. Prior to commencement of the study, the study team, together with community health volunteers, visited the chief of the region and explained the aims and procedures. After acceptance of the study by the chief, durbars (community meetings) were hold, where the work was explained to the communities. For index patient enrolment, the KNUST team worked closely with Mankranso hospital staff. Follow-ups were conducted by a team from KNUST.

### Diagnostic tools

The sensitivities of common malaria diagnostic tools was evaluated, including expert microscopy diagnosis by WHO-certified (Level 1) microscopists from KNUST, Kumasi, field microscopy diagnosis by health care workers in the Mankranso Government Hospital laboratory, RDT diagnosis, and highly sensitive DNA-based *var*ATS quantitative polymerase chain reaction (qPCR) diagnosis. Expert microscopy has long been considered the gold standard for malaria diagnosis. Field microscopy by non-certified microscopists is often used at health posts and often has a lower sensitivity than expert microscopy [12, 13]. Multiple studies have found the sensitivity of RDT diagnosis of *Plasmodium falciparum* to exceed that of field and/or expert microscopy [12, 14]. Results of field microscopy, expert microscopy, and RDTs were compared to qPCR, which is used only for research purposes in Ghana, but not for routine diagnosis due to the requirements for equipment, consumables, and trained laboratory staff.

### Symptomatic case detection in clinical setting

Febrile patients who presented with malaria symptoms to the hospital were eligible to be enrolled. Patients were either admitted to the inpatient department, where they were tested by the local microscopist and tested by RDT by the study team, enabling a comparison of two diagnostic methods. Alternatively, patients were admitted to the outpatient department, where they were only tested by RDT. The decision on whether a patient was referred to the outpatient or inpatient department was taken by the hospital. Patients from both groups were screened by the study team with the same RDT and were eligible to be enrolled in the study. After obtaining informed written consent and completing a brief demographic questionnaire, hospital staff collected a blood sample of approximately 200 µL by venipuncture and recorded body temperature. Blood was collected into an EDTA tube and stored at − 20 °C until DNA extraction.

All study participants were tested by *P. falciparum* RDT Rapigen BIOCREDIT Malaria Ag Pf (pLDH/HRP2, LOT#H052B001D). This test has separate bands for two targets, LDH and HRP2, in addition to the control bands. For each sample, thick and thin film blood smears were prepared in duplicate. At KNUST, expert microscopy was conducted by two independent WHO-certified expert microscopists who were blinded to RDT results and each other’s results. Slides were determined to be positive if parasites were observed to be positive by one or both microscopists.

### Feasibility of reactive intervention programmes

The research team asked all febrile patients presenting to Mankranso Hospital if they would allow a field team to come to their house and test members of their household and neighbouring households by RDT. The research team consisted of scientists from KNUST, which had a long-standing relationship with the hospital, and one foreign scientist. While the research team was not known to patients prior to the study, it was made clear to all patients that the research was conducted in close collaboration with Mankranso hospital staff. For patients who tested positive by RDT, researchers collected phone numbers. Participants who consented to a household visit were contacted via cell phone within 20 days of their visit to the hospital (average 5.21 days). Phone calls were attempted a minimum of three times before a follow-up was counted as unsuccessful. No follow-ups were attempted if the phone was not answered. If participants answered the phone and consented to a follow-up, a field team followed up with the index case household. A neighbour household was defined as a household within 200 m of the index case household, and a control household was defined as any household outside of the 200 m radius. All members of the index case household were invited to participate in the study. For neighbouring and control households, convenience sampling was used to recruit individuals to participate in the study, asking for their house location to determine the household type classification. Households were visited once, and individuals at home were invited to participate. The GPS location of all households was recorded using the iOS compass tool.

Approximately 100 µL of blood was collected by finger prick from each participant into an EDTA tube. The same RDT as for clinical samples was run on-site. Positive individuals were referred to the nearest health centre for treatment. Slides were prepared for expert microscopy at KNUST.

### Molecular diagnosis by DNA-based varATS qPCR testing

Samples were transported frozen to Notre Dame, where DNA extraction was performed with the Macherey-Nagel NucleoMag Blood 200 µL kit. DNA was extracted from 100 µL of whole blood and eluted into 100 µL of elution buffer. Samples were tested for *P. falciparum* using the DNA-based *var*ATS qPCR [15]. This qPCR assay targets *var*ATS, a multicopy gene that has approximately 60 copies per parasite genome, of which approximately 20 are amplified. qPCR was conducted with a total volume of 12 µL, composed of 0.48 µL of 10 µM *var*ATS forward and reverse primers, 0.48 µL 10 µM *var*ATS probe, 6 µL QuantaBio PerfeCTa Tough PCR MasterMix, 1.04 µL H_2_O, and 4 µL eluted DNA. The sensitivity of the extraction and qPCR is approximately 0.3 parasites/µL blood [16]. For absolute quantification, an external standard curve based on cultured *P. falciparum* parasites quantified by digital PCR was run.

### Data analysis

The sensitivity of microscopy and RDT was calculated with qPCR as the most sensitive method for parasite detection. Chi-square tests were run to compare test positivity and prevalence among age groups, sex, and other demographic characteristics.

For RCD analysis, system effectiveness was defined as the proportion of febrile patients correctly diagnosed by field microscopy compared to RDT as the gold standard, multiplied by the proportion of cases willing to be followed up, multiplied by the proportion of successful follow-ups, and multiplied by the proportion of infections diagnosed by RDT compared to qPCR. RDT was used as the gold standard in the hospital as it is the most sensitive diagnostic tool that could realistically be implemented for routine diagnosis. As the goal of RCD programmes is to detect and treat as many subclinical cases as possible, the proportion of infections detected by RDT compared to qPCR was calculated in the last step. The dataset supporting the conclusions of this article is included as Supplementary File S1.

## Results

**Fig. 1:**
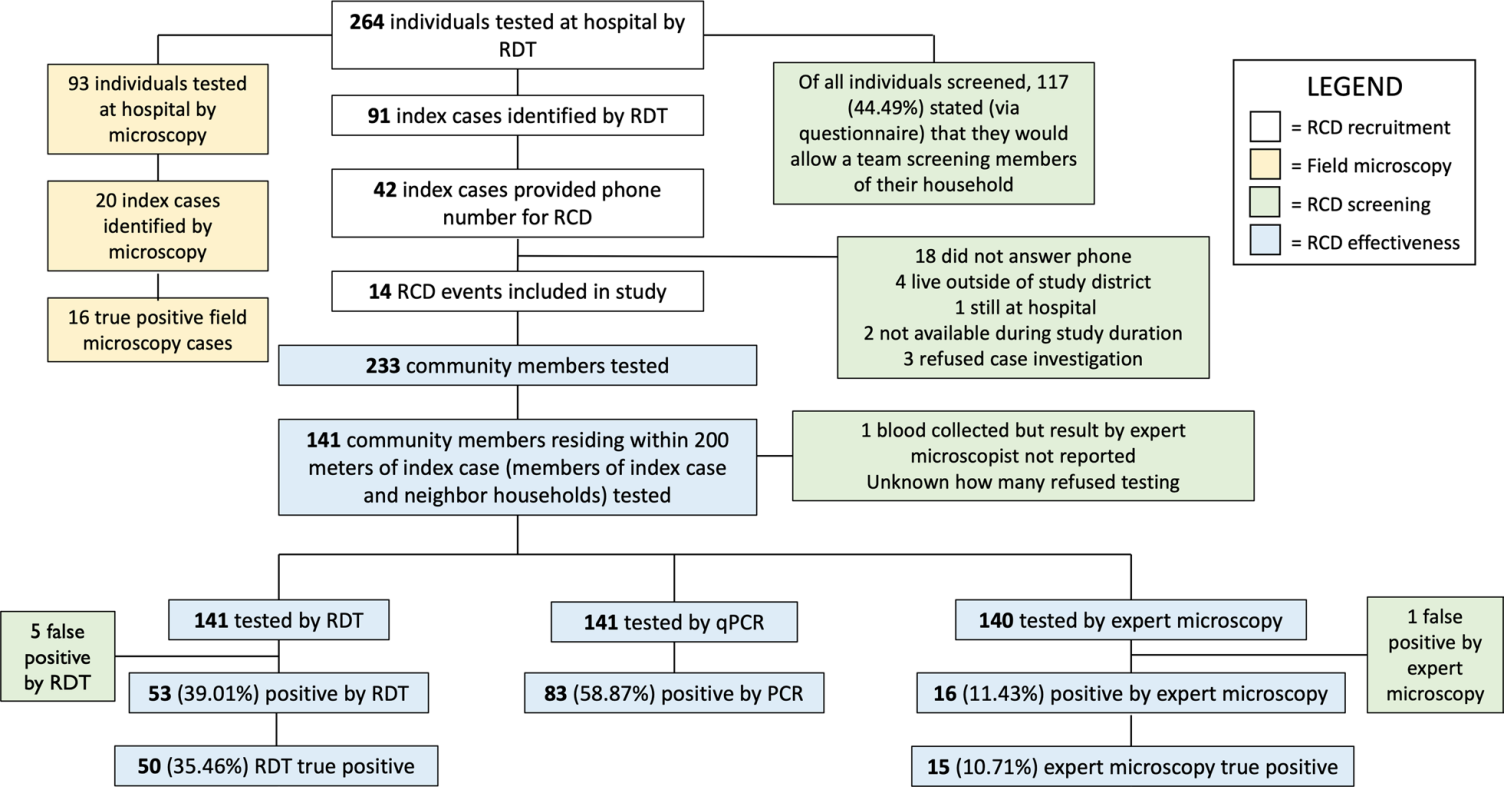
RCD workflow schematic

From June to July 2022, 264 febrile patients were enrolled at the Mankranso Government Hospital, and whole blood samples were collected. 14 RCD follow-ups were conducted, and 233 samples were collected from non-index case RCD participants (Fig. 1). 497 samples were tested by expert microscopy, RDT, and qPCR. Demographic information regarding patient age, sex, travel outside of the individual’s hometown within the last 30 days, insecticide-treated bed net (ITN) usage, and time spent outside after 6 PM is given in Table 1. Individual were asked whether they slept under a bed net the previous night.

**Table 1:** Demographic data for the sample population, including clinical samples (*n* = 264) and household samples (*n* = 233)

	Clinical samples				Household samples			
	N clinical samples	N positive	Positivity by PCR (%)	*P*	N household samples	N positive	Prevalence by PCR	*P*
Age								
< 5	56	26	46.4	0.452	13	7	53.9%	0.002
5–15	37	22	59.5		70	57	81.4%	
> 15	165	88	53.3		150	85	56.7%	
Unspecified	6	3	50.0					
Sex								
Male	87	45	51.7	0.779	100	67	67.0%	0.400
Female	168	90	53.6		133	82	61.7%	
Unspecified	9	4	44.4		0	0	n/a	
Travel								
Yes	57	30	52.6	0.997	39	24	61.5%	0.731
No	207	109	52.7		194	125	64.4%	
Bed net use								
Yes	216	120	55.6	0.036	162	101	64.2%	0.364
No	40	15	37.5		70	48	71.6%	
Unspecified	8	4	50.0		1	0	0%	
Stay outside after								
6:00 PM	53	27	50.9	0.892	46	32	69.6%	0.659
8:00 PM	149	81	54.4		99	63	63.6%	
Later than 10 PM	54	28	51.9		86	53	61.6%	
Unspecified	8	3	37.5		2	1	50.0%	

### Reactive case detection programme implementation

The reactive programme trialed out of the Mankranso Government Hospital presented numerous operational challenges during implementation and execution. Figure 1 shows the workflow of index case recruitment in the clinical setting and attempted follow-ups.

### Test positivity among clinical cases

**Fig. 2:**
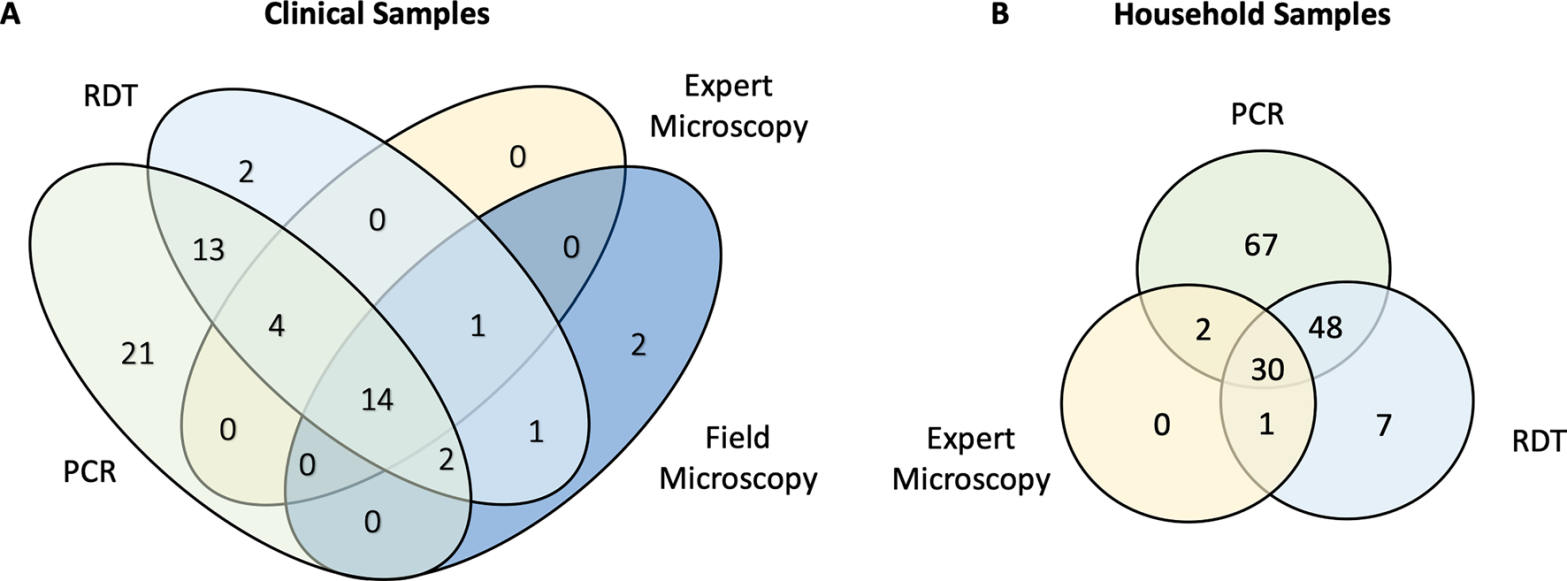
Outcomes of different methods for diagnosis (**A**) Comparison between qPCR, RDT, expert microscopy, and field microscopy for clinical samples tested by all 4 diagnostic methods, including individuals admitted to the inpatient department (*n* = 92), (**B**) Comparison between qPCR, RDT, and expert microscopy for household-based samples tested by 3 diagnostic methods (*n* = 231)

Among 264 febrile patients enrolled, 93 were tested by the microscopist based at the hospital before being referred to the study team for RDT testing. 92 patients were tested by four different diagnostic methods (qPCR, RDT, expert microscopy, and field microscopy, not including one sample for which the expert microscopy result was not recorded, Table 2). Figure 2A illustrates the diagnostic comparisons between the four tests for clinical samples tested by all four tests. RDT detected 33/54 (61.1%) qPCR-positive infections and was approximately twice as sensitive as field microscopy, which detected 16/54 (29.6%) infections. Diagnosis by expert and field microscopy yielded comparable results, both missing a similar proportion of qPCR-positive samples. No significant differences in test positivity were observed between age group, sex, travel status, and time spent outside in the evening (Table 1).

**Table 2:** Diagnostic comparison between qPCR, RDT, expert microscopy, and field microscopy for clinical and household-based samples

Diagnostic tool	*n*	Positivity rate (%)	Sensitivity compared to qPCR	Specificity compared to qPCR	Positive predictive value compared to qPCR
Clinical samples					
qPCR	264	52.65	n/a	n/a	n/a
BioCredit RDT: HRP2/LDH	264	34.47	56.83%	90.48%	86.81%
BioCredit RDT: LDH only	264	21.59	38.85%	97.60%	94.74%
Expert microscopy	263	17.49	31.16%	97.60%	93.48%
Field microscopy	93	21.51	29.09%	89.47%	80.00%
Household samples					
qPCR	233	63.95	n/a	n/a	n/a
BioCredit RDT: HRP2/LDH	233	37.34	53.02%	90.48%	90.80%
BioCredit RDT: LDH only	233	20.60	31.54%	98.81%	97.92%
Expert microscopy	231	14.29	21.77%	98.81%	96.97%

### Prevalence among non-index case RCD participants

80 index case household members, 61 members of neighbouring households, and 92 members of control households were tested by qPCR, RDT, and expert microscopy (Table 3). Overall household prevalence was 63.95% by qPCR, and 37.34% by RDT (Table 2). As seen in clinical case diagnosis results, RDT diagnosis yielded a higher number of positive samples than expert microscopy (Fig. 2B). Compared to qPCR, RDT detected 78/147 (53.06%) infections (Fig. 2B). No difference in prevalence was observed between index case, neighbour, and control households (Table 3). An age trend in prevalence was observed, while sex, travel status, and time spent outside in the evening did not significantly impact prevalence (Table 1).

**Table 3:** Parasite prevalence for different household types by RDT and qPCR diagnosis

Household Type	*n*	Prevalence by RDT (%)	*P*	Prevalence by qPCR (%)	*P*
Index case	80	40.0	0.08	62.50	0.778
Neighbour	61	37.70		54.09	
Control	92	34.78		71.74	

### Reactive case detection programme implementation: effectiveness and operational challenges

The study on the feasibility of reactive interventions operated out of the Mankranso Government Hospital identified numerous operational challenges. Figure 1 exhibits the workflow of index case recruitment in the hospital and follow-ups. The research team screened 264 symptomatic individuals at the Mankranso Government Hospital by RDT. 93 (35.2%) of them were screened by the field microscopist, 20 positive samples were identified, 16 of which were true positives by qPCR. In comparison, RDT detected 33/55 qPCR-positive infections (an additional 4 RDT positive samples were negative by qPCR). RDT diagnosis thus detected a far higher number of index cases than field microscopy. Evidently, the index case recruitment process is vastly impacted by the primary diagnostic method used in the hospital setting.

**Fig. 3:**
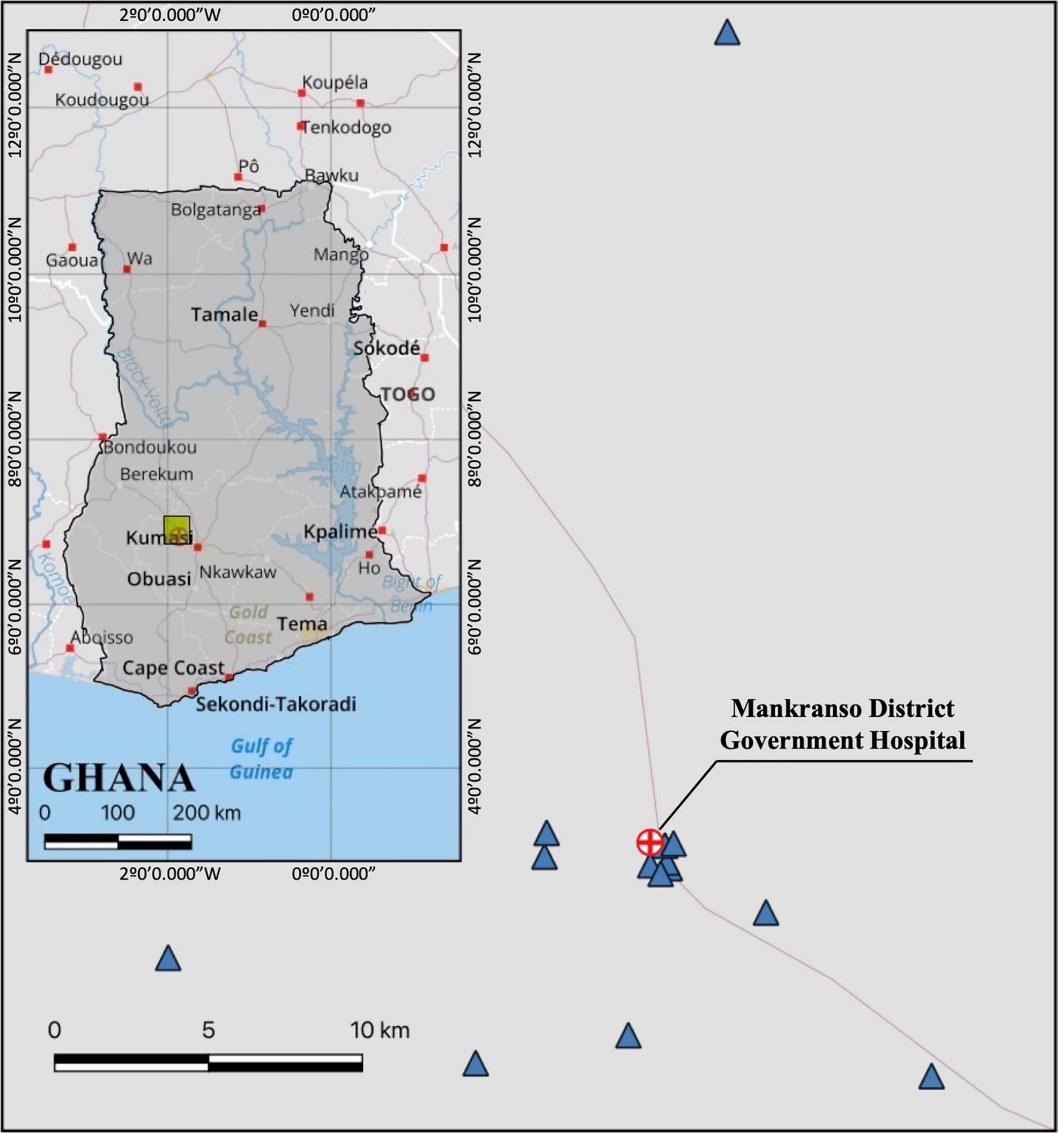
Index case follow-up locations in Ashanti, Ghana

Of all individuals screened, 117/264 (44.5%) stated that they would allow a team to screen members of their household for malaria. There were 91 symptomatic individuals screened by researchers that were positive by RDT (34.5%). All RDT-positive index case individuals (*n* = 91) were asked to provide their phone number to be contacted for the reactive case detection programme, and 42 individuals provided a phone number (*n* = 42/91, 46.1%). Researchers attempted to contact all 42 individuals to execute an RACD follow-up at their households, which led to 14 successful follow-ups (*n* = 14/42, 33.3%). Figure 3 shows the geographic locations of the successful follow-ups.

There were several reasons why the remaining 28 index cases did not lead to successful RACD events. The majority did not answer their phone after repeated attempts to contact them (*n* = 18). The remaining 10 individuals were contacted but follow-ups could not be executed for the following reasons: four individuals lived outside the area of study jurisdiction, two individuals were not available for a household visit during the study duration, one individual remained at the hospital in the inpatient department for the study duration, and three individuals refused case investigation upon further contact with researchers via phone call.

Researchers were interrupted while screening asymptomatic individuals at two RACD events due to backlash from community members. In one rural index case setting, a local religious leader was suspicious of the researchers’ intentions and study authorization. He asked researchers to leave after screening 12 individuals. At another index case household, a community member questioned the study’s purpose and refused to participate, convincing many others to do the same. For all follow-ups, the number of individuals who were not at home when the RACD screening was done was not recorded. Across all households, RDT detected 79/149 (53.0%) of infections positive by qPCR (Fig. 1E).

### Reactive case detection programme effectiveness and operational challenges

**Fig. 4:**
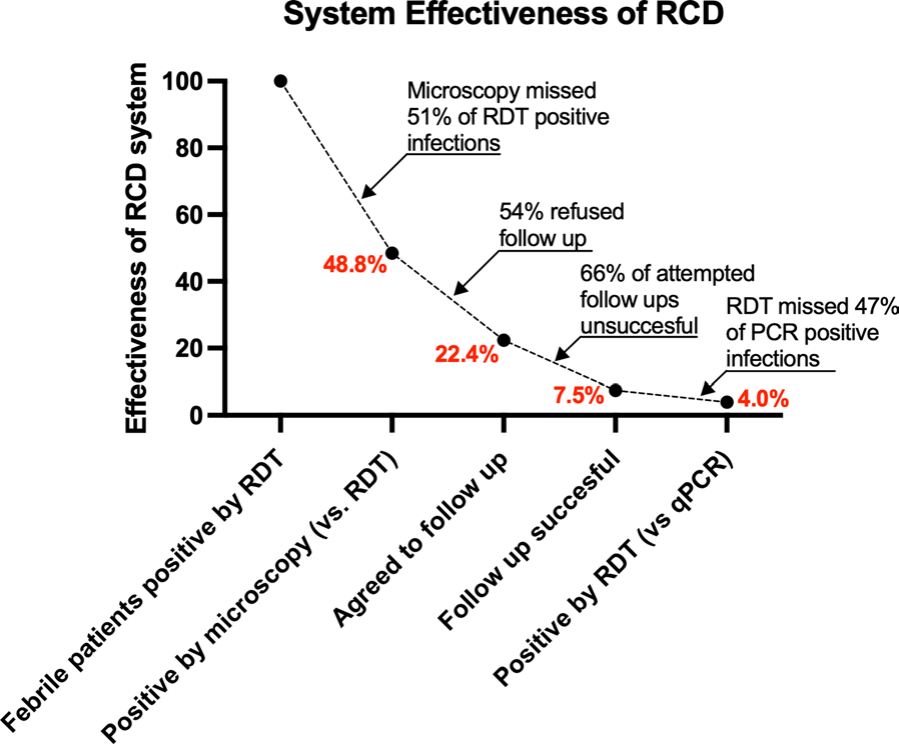
Potential RCD programme system effectiveness

The effectiveness of the RCD system synthesizes operational challenges caused by low diagnostic sensitivities and low community receptivity (Fig. 4). The system effectiveness drops due to the infections missed due to low field microscopy sensitivity compared to RDT. After the diagnostic challenges at the health centre, index cases that refuse follow-ups (54%) and unsuccessful attempted follow-ups (66%) lead to a further drop in system effectiveness. Finally, low RDT sensitivity among non-index case RCD participants tested during RCD events leads to an overall system effectiveness of 4.0%.

## Discussion

In this study, the sensitivity of different tools for malaria diagnosis were compared among clinical and subclinical individuals, and the operation challenges and feasibility of a future reactive interventions was investigated. Transmission in this region is high, but reactive interventions with operational improvements may become a viable and necessary option for malaria control once transmission is reduced and pre-elimination status is achieved in several districts as aimed for by NMCP [9].

Two major limitations to a successful RCD programme in Ghana’s Ashanti region were identified: the low diagnostic sensitivity for symptomatic and asymptomatic infections by routine microscopy, and low receptivity to RCD-style follow-ups among study participants. Together, these factors resulted in a potential system effectiveness of 4.0% in this study, meaning that an RCD programme effectively detected only 4.0% of the asymptomatic qPCR-positive infections that a perfectly effective system would hypothetically detect.

The low sensitivity of the microscopic diagnosis currently applied in the hospital could be resolved by the introduction of novel, highly sensitive RDTs like the one used in this study. This RDT still missed over 40% of qPCR positive infections. While it is impossible for any single infection to determine whether it is the cause of febrile illness, it is likely that many of these low-density infections were not the cause of fever but reflect the high prevalence of infection in the study population. Thus, they do not represent true index cases and do not need to be followed up.

Next, over half of index cases did not wish to be followed up, and two thirds of attempted follow ups were unsuccessful. While the Ghanian researchers who spoke to study participants had a long-standing relationship with the hospital, they were not members of the greater Mankranso community and therefore were not personally known to the community members, which may have negatively impacted community receptivity. Once a routine reactive intervention system was introduced, familiarity with the process and the health officials who conduct the follow-ups is expected to increase receptivity to follow-up events. This is evidenced by current IRS programmes in Ghana. Communities are generally receptive to IRS conducted by the NMCP [9]. Also, a study about the receptivity to a preventative SMC (seasonal malaria chemopreventative) programme in Ghana found that caregivers’ trust in and respect for the administrators of SMC helped increase their level of uptake of SMC [17]. The NMCP already plans to increase uptake and acceptance of malaria intervention programmes including community action plans developed by community health management committees, door-to-door education visits by community health officers and volunteers, and education sessions at mosques and churches on malaria prevention [9].

Of note, even in countries where RCD is routinely conducted, following up index cases is a challenge. In Zambia, only 32% of eligible index cases were followed up, and in the households followed up only 66% of residents were at home [18]. The Zanzibar Malaria Elimination Programme has implemented an island-wide reactive case detection programme since 2012. Yet, a study found that only 35% of follow ups were successful within 3 days [19]. Different reasons caused unsuccessful follow ups, including lack of RDTs in Zambia and failure to report index cases to the control programme or failure to follow up cases in Zanzibar. It is not known to what extent unwillingness of index cases to provide contact data prevented successful follow ups.

Lastly, RDTs missed 47% of qPCR positive subclinical infections. In potential future reactive intervention programmes, reactive focal drug administration (rFDA) should be considered as a viable and effective alternative. Evidence from previous studies points to rFDA as a more effective, quicker, and feasible reactive intervention programme in low transmission settings as compared to RCD [6, 7].

Several factors contributing to RCD system effectiveness were not assessed in this study. Treatment seeking was not investigated; thus, it is not known what proportion of potential index cases did not present to the hospital. The study team contacted individuals multiple times by phone and only attempted follow-ups once they got confirmation that people were at home. Data from this study cannot directly be compared to programmes that collect addresses at enrolment, and then conduct follow-ups without contacting index cases by phone. For the current study, the team returned to each household only once. As a result, individuals not at home during the visit were not tested, and the number of individuals not at home during follow-ups was not determined. Individuals not present for testing further reduce system effectiveness. On the other hand, the study did not face challenges that routine reactive intervention programmes have faced. Follow-ups were conducted by a research team using a KNUST vehicle. The team also brought the RDTs required for testing. For a routine programme, maintenance of the car and lack of RDTs were reported to be challenges [18]. The average time between hospital visit and RCD follow-up was 5.21 days, yet there is evidence that individuals tested within 3 days of the index case diagnosis are more likely to test positive [20]. It is not known whether the window of time between index case notification and RCD testing would impact the prevalence in households.

Due to the endemic nature of the disease setting, at present, targeted interventions are currently not recommended. Once prevalence levels in Ghana decline, reactive interventions such as RCD or rFDA might become a feasible and more resource efficient option to shrink the asymptomatic reservoir. Follow up of clinical patients can be beneficial for surveillance beyond the reduction of the reservoir of subclinical infections. Reactive household visits might serve to monitor the utilization and quality of bed bets and adoption of other vector-control interventions [21], to understand attitudes towards treatment seeking [22]. Trials have shown that such visits can result in individuals adopting better protection against malaria [23]. In case of suspected treatment failures identified at health centres (e.g., the same patient presenting with parasitaemia again shorty after treatment), reactive sample collection followed by sequencing of markers of drug resistance can be conducted to identify potential clusters of drug resistant parasites. The RDT used in this study, with separate lines for HRP2 and LDH, allows phenotypic identification of possible *hrp2*/*3* deletions. If deletions are detected, household follow-ups to diagnose and genotype subclinical infections might be warranted. In conclusion, the operational challenges identified through this study may help in the design of such surveillance and reactive intervention programmes in this area.

## Supplementary Information

Supplementary Material 1: Database

## Data Availability

The dataset supporting the conclusions of this article is included as Supplementary File S1.

## Abbreviations

CHPS: Community-based Health Planning and Services
HRP2: Histidine rich protein 2
IPTp: intermittent preventative treatment in pregnancy
ITN: Insecticide-treated net
LDH: Lactate dehydrogenase
MDA: Mass drug administration
MTAT: Mass test and treat
qPCR: Quantitative polymerase chain reaction
RCD: Reactive case detection
RDT: Rapid diagnostic test
rFDA: Reactive focal drug administration
WHO: World Health Organization

## References

[1] WHO. World malaria report 2022. Geneva: World Health Organization; 2022.

[2] Rek J, Blanken SL, Okoth J, Ayo D, Onyige I, Musasizi E, et al. Asymptomatic school-aged children are important drivers of malaria transmission in a high endemicity setting in Uganda. J Infect Dis. 2022;226:708–13. 35578987 10.1093/infdis/jiac169 PMC9441202

[3] Andolina C, Rek JC, Briggs J, Okoth J, Musiime A, Ramjith J, et al. Sources of persistent malaria transmission in a setting with effective malaria control in eastern Uganda: a longitudinal, observational cohort study. Lancet Infect Dis. 2021;21:1568–78. 34146476 10.1016/S1473-3099(21)00072-4 PMC8554388

[4] van Eijk AM, Ramanathapuram L, Sutton PL, Kanagaraj D, Sri Lakshmi Priya G, Ravishankaran S, et al. What is the value of reactive case detection in malaria control? A case-study in India and a systematic review. Malar J. 2016;15:67. 26852118 10.1186/s12936-016-1120-1 PMC4744450

[5] Stresman G, Whittaker C, Slater HC, Bousema T, Cook J. Quantifying *Plasmodium falciparum* infections clustering within households to inform household-based intervention strategies for malaria control programs: an observational study and meta-analysis from 41 malaria-endemic countries. PLoS Med. 2020;17:e1003370. 33119589 10.1371/journal.pmed.1003370 PMC7595326

[6] Bridges DJ, Miller JM, Chalwe V, Moonga H, Hamainza B, Steketee RW, et al. Reactive focal drug administration associated with decreased malaria transmission in an elimination setting: serological evidence from the cluster-randomized CoRE study. PLoS Glob Public Health. 2022;2:e0001295. 36962857 10.1371/journal.pgph.0001295 PMC10021141

[7] Hsiang MS, Ntuku H, Roberts KW, Dufour M-SK, Whittemore B, Tambo M, et al. Effectiveness of reactive focal mass drug administration and reactive focal vector control to reduce malaria transmission in the low malaria-endemic setting of Namibia: a cluster-randomised controlled, open-label, two-by-two factorial design trial. Lancet. 2020;395:1361–73. 32334702 10.1016/S0140-6736(20)30470-0 PMC7184675

[8] WHO. Recommendations on malaria elimination. Geneva: World Health Organization; 2021.

[9] U.S. President’s Malaria Initiative Ghana. Malaria operational plan FY 2022. https://www.pmi.gov/. Accessed 5 May 2024.

[10] Ghana Statistical Service. Ghana Malaria Indicator Survey 2019. Accra, Ghana; 2020.

[11] Heinemann M, Phillips RO, Vinnemeier CD, Rolling CC, Tannich E, Rolling T. High prevalence of asymptomatic malaria infections in adults, Ashanti Region, Ghana, 2018. Malar J. 2020;19:366. 33046056 10.1186/s12936-020-03441-z PMC7552528

[12] Afriyie SO, Addison TK, Gebre Y, Mutala A-H, Antwi KB, Abbas DA, et al. Accuracy of diagnosis among clinical malaria patients: comparing microscopy, RDT and a highly sensitive quantitative PCR looking at the implications for submicroscopic infections. Malar J. 2023;22:76. 36870966 10.1186/s12936-023-04506-5 PMC9985253

[13] Coleman RE, Maneechai N, Rachaphaew N, Kumpitak C, Miller RS, Soyseng V, et al. Comparison of field and expert laboratory microscopy for active surveillance for asymptomatic *Plasmodium falciparum* and *Plasmodium Vivax* in western Thailand. Am J Trop Med Hyg. 2002;67:141–4. 12389937 10.4269/ajtmh.2002.67.141

[14] Mfuh KO, Achonduh-Atijegbe OA, Bekindaka ON, Esemu LF, Mbakop CD, Gandhi K, et al. A comparison of thick-film microscopy, rapid diagnostic test, and polymerase chain reaction for accurate diagnosis of *Plasmodium Falciparum* malaria. Malar J. 2019;18:73. 30866947 10.1186/s12936-019-2711-4 PMC6416847

[15] Hofmann N, Mwingira F, Shekalaghe S, Robinson LJ, Mueller I, Felger I. Ultra-sensitive detection of *Plasmodium falciparum* by amplification of multi-copy subtelomeric targets. PLoS Med. 2015;12:e1001788. 25734259 10.1371/journal.pmed.1001788 PMC4348198

[16] Holzschuh A, Koepfli C. Tenfold difference in DNA recovery rate: systematic comparison of whole blood vs. dried blood spot sample collection for malaria molecular surveillance. Malar J. 2022;21:88. 35292038 10.1186/s12936-022-04122-9 PMC8922754

[17] Antwi GD, Bates LA, King R, Mahama PR, Tagbor H, Cairns M, et al. Facilitators and barriers to uptake of an extended seasonal malaria chemoprevention programme in Ghana: a qualitative study of caregivers and community health workers. PLoS ONE. 2016;11:e0166951. 27898699 10.1371/journal.pone.0166951 PMC5127521

[18] Searle KM, Hamapumbu H, Lubinda J, Shields TM, Pinchoff J, Kobayashi T, et al. Evaluation of the operational challenges in implementing reactive screen-and-treat and implications of reactive case detection strategies for malaria elimination in a region of low transmission in southern Zambia. Malar J. 2016;15:412. 27527347 10.1186/s12936-016-1460-x PMC4986207

[19] van der Horst T, Al-Mafazy AW, Fakih BS, Stuck L, Ali A, Yukich J, Hetzel MW. Operational coverage and timeliness of reactive case detection for malaria elimination in Zanzibar, Tanzania. Am J Trop Med Hyg. 2019;102:298–306. 10.4269/ajtmh.19-0505 PMC7008315 31769395

[20] Stuck L, Fakih BS, Al-Mafazy AH, Hofmann NE, Holzschuh A, Grossenbacher B, et al. Malaria infection prevalence and sensitivity of reactive case detection in Zanzibar. Int J Infect Dis. 2020;97:337–46. 32534138 10.1016/j.ijid.2020.06.017 PMC8450816

[21] Lobo NF, Achee NL, Greico J, Collins FH. Modern vector control. Cold Spring Harb Perspect Med. 2018;8:a025643. 28507198 10.1101/cshperspect.a025643 PMC5749144

[22] Dixit A, Lee M-C, Goettsch B, Afrane Y, Githeko AK, Yan G. Discovering the cost of care: consumer, provider, and retailer surveys shed light on the determinants of malaria health-seeking behaviours. Malar J. 2016;15:179. 27006074 10.1186/s12936-016-1232-7 PMC4802645

[23] Bekolo CE, Williams TD. Adding proactive and reactive case detection into the integrated community case management system to optimise diagnosis and treatment of malaria in a high transmission setting of Cameroon: an observational quality improvement study. BMJ Open. 2019;9:e026678. 31182444 10.1136/bmjopen-2018-026678 PMC6561439

